# Community health workers’ knowledge and attitudes toward advance directives in China: a cross-sectional study

**DOI:** 10.3389/fpubh.2025.1661934

**Published:** 2025-09-12

**Authors:** Jianshu Zhang, Ronghua Fang

**Affiliations:** General Practice Ward, International Medical Center Ward, General Practice Medical Center, West China Hospital/West China School of Nursing, Sichuan University, Chengdu, China

**Keywords:** community health workers, advance directives, knowledge, attitudes, cross-sectional study

## Abstract

**Background:**

Advance Directives (ADs), including living wills and durable powers of attorney for healthcare, are vital to uphold patient autonomy in end-of-life care, reducing family conflicts, shortening hospital stays. ADs’ implementation varies globally. In China, both the public and healthcare workers have a relatively low awareness of ADs. Community health workers (CHWs) play a significant role in the quality of end-of-life care, but there is a lack of relevant research in China. This study aims to explore Chinese CHWs’ knowledge, attitudes, and intentions regarding ADs and identifies influencing factors to inform policy recommendations for ADs implementation in China.

**Methods:**

A cross-sectional survey was conducted among 426 CHWs from 12 community health centers in southwestern China (December 2024–February 2025) via online questionnaires. Data included demographics, ADs knowledge, intentions, and attitudes. SPSS 21.0 was used for chi-square tests, t-tests, and binary logistic regression to analyze influencing factors.

**Results:**

Among 426 CHWs, only 22.54% possessed prior knowledge of ADs, with a mean knowledge score of 3.82 ± 2.85. Although 83.33% expressed support for ADs, merely 46.95% reported willingness to sign one. The predominant barrier identified by 87.79% of CHWs was the lack of relevant legislation. Logistic regression analysis revealed that: (1) each one-point increase in ADs knowledge score was associated with a 25% higher likelihood of supporting ADs (OR = 1.250, *p* < 0.05); (2) CHWs with higher education levels were significantly more likely to support ADs (OR = 1.547, *p* < 0.05); and (3) CHWs’ belief in participating in medical decision-making was negatively correlated with their level of ADs support (OR = 0.386, *p* < 0.05).

**Conclusion:**

CHWs in China exhibit limited knowledge of ADs but generally supportive attitudes. However, the gap between expressed support and personal willingness to sign ADs, coupled with the perceived legal barrier, highlights an urgent need for targeted interventions to facilitate ADs implementation. Key policy priorities should include establishing a legal framework for ADs, clarifying the role of CHWs in end-of-life care processes, and incorporating mandatory ADs training into continuing medical education programs.

## Introduction

Advance Directives (ADs), encompassing living wills (LWs) and durable powers of attorney for healthcare (1, 2), serve as indispensable instruments for upholding patient autonomy in end-of-life (EOL) care decisions. Substantial evidence demonstrates that ADs reduce family conflicts in medical decision-making (3), shorten hospital stays (4), decrease non-beneficial life-sustaining treatments (5), and promote hospice utilization (6). These outcomes establish ADs as a cornerstone of patient-centered care models.

Global ADs implementation highlights significant disparities influenced by cultural norms and systemic barriers. Research shows that in East Asian countries like Japan, Hong Kong, and South Korea, there is low adoption due to cultural barriers (7). In Portugal, despite more than a decade of established legal frameworks, the implementation of ADs remains limited (8). In Germany, ADs are underutilized due to insufficient public awareness (9). Similarly, India and Spain grapple with exceedingly low utilization rates, notwithstanding the enactment of relevant legislation (10, 11). In contrast, awareness campaigns in the United States and Switzerland have drawn attention to racial/ethnic disparities and professional skepticism regarding ADs (6, 12). Moreover, research indicates that in China, both the general public and healthcare professionals have low awareness levels of ADs (13).

This implementation gap underscores the crucial role of community health workers (CHWs) as frontline intermediaries between healthcare systems and vulnerable populations. Endowed with competencies in health literacy promotion and chronic disease management (14, 15), CHWs are well-positioned to act as catalysts for ADs adoption. Grounded in the KAP (Knowledge, Attitude, and Practice) theory, which posits that knowledge shapes attitudes, which in turn influence behaviors (16), the knowledge base and attitudes of CHWs toward ADs are not only instrumental in their own AD practices but also significantly impact the quality of care they deliver to patients (17, 18). Previous surveys of healthcare professionals in Germany have identified inadequate understanding and a lack of proactive engagement with ADs as the primary barriers to their implementation (19). Research from Singapore has also highlighted the need for further training, as CHWs often grapple with conceptual misunderstandings of ADs (20).

Notably, there is a dearth of research focusing specifically on CHWs’ perspectives on ADs in China. To address this gap, the present cross-sectional study aims to assess the knowledge and attitudes of CHWs in China regarding ADs, identify the barriers and facilitators influencing their knowledge and attitudes, explore the potential of CHWs as advocates for ADs in alignment with the global shift toward patient-centered care models, and provide policymakers with evidence-based insights to reduce disparities in access to dignified EOL care.

## Materials and methods

### Study design and participants

This study conducted a multi-center cross-sectional study across 12 community health centers in Sichuan Province in southwestern China, employing convenience sampling to capture real-world CHWs’ preparedness for ADs advocacy. The inclusion criteria were as follows: (1) certified CHWs with ≥1 year community service experience; (2) agreement to participate in this survey. The exclusion criteria were: (1) administrative staff without clinical duties; (2) leave status exceeding 30 days during study period. The data were collected between December 2024 and February 2025, a questionnaire link and QR code were distributed to CHWs who met the inclusion and exclusion criteria for the sample.

### Sample size calculations

The sample size was calculated using the formula for cross-sectional studies:

*n* = Z_*α*/2_^2^*π*(1 − *π*)/*d*^2^. When *α* = 0.05, *Z*_α/2_ = 1.96, the assumed degree of variability (*π* = 0.5) was used to maximize the required sample size, and *d* (admissible error) was set at 5%. The theoretical sample size was calculated to be 385.

### Measurement

Four distinct questionnaires were disseminated to participants via links and quick response (QR) codes, enabling them to complete and submit the surveys online. This digital approach ensured convenience and streamlined data collection. The questionnaires were pilot tested with 20 CHWs in order to revise it for well understanding and clarity.

Demographic information and the respondents’ personal values were collected by a self-designed questionnaire. It encompasses gender, marital status, work-related information, as well as the respondent’s values, disease history, experience with hospital training on ADs, and exposure to terminal situations. This comprehensive data collection aimed to provide a detailed profile of the participants, which could potentially influence their knowledge, attitudes, and intentions regarding ADs.

CHWs’ knowledge of ADs was evaluated using the ADs knowledge scale designed by researchers in Hong Kong (21). The scale assesses respondents’ understanding of ADs, encompassing aspects such as content, format, and applicable scenarios. To minimize response bias stemming from guessing, response options were presented as “yes,” “no,” and “do not know.” During scoring, a correct answer was assigned 1 point, while incorrect answers or “do not know” responses received 0 points. Comprising 10 items, the scale yields a total score ranging from 0 to 10. In this research, the Cronbach’s *α* coefficient of the scale was 0.928, indicating a high level of internal consistency.

CHWs’ intentions regarding ADs were assessed using a comprehensive advance directives questionnaire (22). This instrument, initially developed as a medical document by Emannule and Emannule (23), underwent translation into Chinese and cultural adaptation by Zhang et al. (22). It includes participants’ willingness to sign ADs, appoint a medical proxy, donate organs and their preferences for specific treatments in four EOL scenarios: a possibility of recovery for coma, persistent vegetative state, dementia, dementia at the terminal stage of a disease. For each scenario, CHWs were required to articulate their preferences regarding life-sustaining interventions.

CHWs’ attitudes toward ADs were evaluated using the questionnaire developed by Zhang et al. (22). It consisted of 50 items and comprehensively explored respondents’ subjective perceptions of patients’ end-stage conditions, their understanding of patient autonomy, the impact of the patient’s family, and concerns about potential abuse. The responses were coded in a binary format (yes = 1, no = 0). In this study, the Cronbach’s *α* coefficient of this questionnaire reached 0.959, signifying excellent internal reliability.

### Statistical analysis

Statistical analyses were conducted using SPSS software (Version 21.0, IBM Corporation, Armonk, NY, USA). The sample distribution was first verified to conform to the normal distribution. Sample characteristics were analyzed using descriptive statistics. Continuous variables were presented as the mean and standard deviation, while categorical variables were described using frequencies and percentages. To examine associations within the general information and knowledge components, Chi-square tests and t-tests were performed. Binary logistic regression analysis was employed to determine the influence of various factors on CHWs’ attitudes toward ADs. A *p*-value <0.05 was considered significant.

## Results

### Demographic data and values

A total of 450 CHWs who met the inclusion and exclusion criteria of the sample participated in the survey. Among them, there were 426 valid questionnaires were collected, yielding an effective response rate of 94.7%.

The sample exhibited the following characteristics: there were 114 doctors (26.76%), ranged in age from 22 to 64 years old, the average age was 35.87 ± 8.42. 377 out of 426 were female (88.5%). 420 (98.59%) were Han Chinese. 272 individuals (63.85%) held a bachelor’s degree, and 434 (80.52%) were married. Further characteristics are detailed in Table 1. In terms of issues related to medical experiences, 170 (39.91%) of the sample believed that their health condition was average, 388 (91.08%) CHWs stated that they had not suffered from any diseases that seriously affected their life and work, while 230 (53.99%) CHWs had an experience of relatives’ painful death. As in values, 307 (72.07%) out of 426 participants did not think that discussing life and death discomfort them and 313 (73.47%) CHWs wanted to participate in their own medical decisions. Although 189 (44.37%) of the sample believed humans can control their own destiny, only 84 (19.72%) CHWs thought that the length of life was more important than the quality of life. 319 medical staff reported that community health center where they work lacked ethics committee, and only 22.54% of the participants have heard of ADs before. Additional details are presented in Table 2.

**Table 1:** Demographic data of participants (*n* = 426).

Variables	*n* (%)
Gender	
Male	49 (11.50)
Female	377 (88.50)
Age, y	
≤30	134 (31.46)
31–40	184 (43.19)
41–50	82 (19.25)
≥51	26 (6.10)
Ethnicity	
Han	420 (98.59)
Minority	6 (1.41)
Occupation	
Nurse	312 (73.24)
Doctor	114 (26.76)
Education level	
Secondary technical school	19 (4.46)
Junior college	131 (30.75)
Bachelor’s degree	272 (63.85)
Master’s degree or above	4 (0.94)
Professional title	
None	79 (18.54)
Junior	143 (33.57)
Intermediate	166 (38.97)
Associate senior	27 (6.34)
Senior	11 (2.58)
Manager position	
None	369 (86.62)
Yes	57 (13.38)
Work experience, y	
<1	7 (1.64)
1–5	66 (15.49)
6–10	105 (24.65)
11–15	114 (26.76)
16–20	52 (12.21)
>20	82 (19.25)
Monthly income (Yuan)	
<3,000	35 (8.22)
3,000–5,000	227 (53.29)
5,001–10,000	153 (35.92)
>10,000	11 (2.58)
Marital status	
Unmarried	77 (18.08)
Married	343 (80.52)
Divorced	6 (1.41)
Living status	
Living alone	35 (8.22)
Living with family	378 (88.73)
Living with non-family	13 (3.05)
Number of children	
None	105 (24.65)
One	231 (54.23)
Two or more	90 (21.13)
Religious belief	
Yes	5 (1.17)
No	421 (98.83)

**Table 2:** Values and medical experiences among CHWs (*n* = 426).

Variables	*n* (%)
Self-rated health	
Very good	103 (24.18)
Good	141 (33.1)
Average	170 (39.91)
Poor	9 (2.11)
Very poor	3 (0.70)
History of serious illness affecting work/life	
Yes	38 (8.92)
No	388 (91.08)
Experience of relatives’ painful death	
Yes	230 (53.99)
No	196 (46.01)
Discussing life and death discomfort you	
Yes	119 (27.93)
No	307 (72.07)
Belief in participating in medical decision-making	
Yes	313 (73.47)
No	113 (26.53)
Views on destiny	
Humans can control their destiny	189 (44.37)
Do not want to plan ahead, wait and see	116 (27.23)
Humans cannot change what will happen	121 (28.40)
Difficulty in making medical decisions for self vs. seriously ill relatives	
Self	132 (30.99)
Relatives	294 (69.01)
Importance of quality of life vs. length of life	
Quality of life is more important	342 (80.28)
Length of life is more important	84 (19.72)
Presence of ethics committee in hospital	
Yes	107 (25.12)
No	319 (74.88)
Experience with terminal patients	
Yes	291 (68.31)
No	135 (31.69)
Discussed terminal medical choices with patients	
Yes	102 (23.94)
No	324 (76.06)
Experience with prolonged treatment of unconscious terminal patients due to family’s reluctance to give up	
Yes	265 (62.21)
No	161 (37.79)
Experience with family disputes over patient treatment choices	
Yes	281 (65.96)
No	145 (34.04)
Considered own treatment choices in terminal illness	
Yes	201 (47.18)
No	225 (52.82)
Heard of advance directives before	
Yes	96 (22.54)
No	330 (77.46)
Impact of ADs on terminal treatment aspects	
(1) Treatment measures	
No impact	73 (17.14)
Impact	353 (82.86)
(2) Pain	
No impact	110 (25.82)
Impact	316 (74.18)
(3) Terminal illness duration	
No impact	113 (26.53)
Impact	313 (73.47)
Has your hospital provided any training on ADs?	
Yes	65 (15.26)
No	361 (84.74)
What kind of institution do you think should undertake the training for ADs?	
University	69 (16.20)
Hospital	255 (59.86)
Academic organization	102 (23.94)

### CHWs’ knowledge level of ADs

The average knowledge score of participants was 3.82 ± 2.85. There was no statistically significant difference in the scores between doctors and nurses (Table 3).

**Table 3:** Comparison of knowledge level of ADs between doctors and nurses.

Items	Occupation		*t*	*p*
	Nurses (*n* = 312)	Doctors (*n* = 114)		
Only the older adults (aged 60 or above) are permitted to make advance directives.	0.32 ± 0.47	0.41 ± 0.49	−1.782	0.076
Only when the patient fully understands the meaning of the medical condition in the advance directive can it be regarded as valid.	0.52 ± 0.50	0.57 ± 0.50	−0.876	0.382
Carrying out advance directive is another form of euthanasia.	0.21 ± 0.41	0.30 ± 0.46	−1.774	0.078
Advance directives allow for refusal of life-sustaining treatment.	0.48 ± 0.50	0.55 ± 0.50	−1.313	0.190
The advance directive must be dated and signed by two witnesses, at least one of whom should be a doctor.	0.61 ± 0.49	0.61 ± 0.49	−0.095	0.925
Dementia is the most common scenario for advance directives.	0.43 ± 0.50	0.43 ± 0.50	0.053	0.958
Once an advance directive is made, it cannot be revoked.	0.45 ± 0.50	0.46 ± 0.50	−0.297	0.767
Advance directives can only be activated when the patient reaches the terminal stage or falls into an irreversible coma.	0.14 ± 0.35	0.15 ± 0.36	−0.126	0.899
Advance directives can be either in written form or in oral form.	0.21 ± 0.41	0.26 ± 0.44	−1.088	0.278
Advance directives include living wills and durable power of attorney for healthcare.	0.48 ± 0.50	0.56 ± 0.50	−1.415	0.158

### CHWs’ intentions regarding ADs

Among the participants, 200 (46.95%) CHWs indicated that they would sign an AD if it was legal, while 135 (31.69%) CHWs did not know whether they should sign an AD, and the rest of them chose not to sign an AD. 294 (69.01%) CHWs would be willing to designate a medical proxy if they need. When it came to whether to donate organs, 205 (48.12%) of the samples would like to donate organs after their death and 132 (64.39%) of them would donate any organs.

The intentions of CHWs regarding the acceptance or rejection of certain treatment measures varied across four terminal scenarios. In the scenario “a possibility of recovery from coma,” the measures that ranked in the top five in terms of acceptance were routine examinations (86.62%), minor surgery (77.70%), antibiotics (77.23%), cardiopulmonary resuscitation (CPR, 74.41%) and major surgery (72.30%). In contrast, in the scenario “persistent vegetative state,” the top five refused measures were major surgery (80.05%), CPR (77.93%), mechanical ventilation (72.30%), minor surgery (67.37%) and invasive procedures (63.85%). The medical decision-making of scenario “dementia” presented the characteristic of priority of comfort treatment: the top five acceptance rates were of analgesics (69.95%), routine examinations (59.62%), antibiotics (49.53%), artificial nutrition (46.71%) and blood transfusion or blood products (23.94%). The refusal rate for scenario “dementia at the terminal stage of a disease” was extremely high: the top five refusal rates were CPR (89.44%), mechanical ventilation (84.98%), chemotherapy (84.74%), invasive procedures (83.10%) and minor surgery (82.86%) (Table 4).

**Table 4:** CHWs’ choices at four terminal scenarios (n, %).

Variables	A possibility of recovery for coma				Persistent vegetative state				Dementia				Dementia at the terminal stage of a disease			
	Yes	No	Try	Uncertain	Yes	No	Try	Uncertain	Yes	No	Try	Uncertain	Yes	No	Try	Uncertain
Cardiopulmonary resuscitation (CPR)	317 (74.41)	75 (17.61)	/	34 (7.98)	74 (17.37)	332 (77.93)	/	20 (4.70)	95 (22.30)	286 (67.13)	/	45 (10.57)	33 (7.75)	381 (89.44)	/	12 (2.81)
Mechanical ventilation	256 (60.09)	91 (21.36)	37 (8.69)	42 (9.86)	90 (21.13)	308 (72.30)	8 (1.88)	20 (4.72)	92 (21.60)	290 (68.08)	11 (2.58)	33 (7.74)	32 (7.51)	362 (84.98)	2 (0.47)	30 (7.04)
Artificial nutrition	287 (67.37)	56 (13.15)	42 (9.86)	41 (9.62)	201 (47.18)	192 (45.07)	10 (2.35)	23 (5.40)	199 (46.71)	197 (46.24)	5 (1.17)	25 (5.88)	61 (14.32)	352 (82.63)	2 (0.47)	11 (2.58)
Major surgery (e.g., gallbladder or intestinal resection)	308 (72.30)	63 (14.79)	/	55 (12.91)	67 (15.73)	341 (80.05)	/	18 (4.22)	88 (20.66)	277 (65.02)	/	43 (14.32)	35 (8.22)	349 (81.92)	/	42 (9.86)
Renal dialysis	217 (50.94)	95 (22.30)	48 (11.27)	66 (15.49)	99 (23.24)	263 (61.74)	14 (3.28)	50 (11.74)	101 (23.71)	252 (59.15)	31 (7.28)	42 (9.86)	54 (12.68)	337 (79.11)	23 (5.40)	12 (2.81)
Chemotherapy	193 (45.31)	114 (26.76)	51 (11.97)	68 (15.96)	121 (28.40)	235 (55.16)	12 (2.82)	58 (13.62)	78 (18.31)	277 (65.02)	39 (9.15)	32 (7.52)	26 (6.10)	361 (84.74)	4 (0.94)	35 (8.22)
Minor surgery (e.g., amputation of infected toe)	331 (77.70)	38 (8.92)	/	57 (13.38)	89 (20.89)	287 (67.37)	/	50 (11.74)	45 (10.56)	330 (77.46)	/	51 (11.98)	31 (7.28)	353 (82.86)	/	42 (9.86)
Invasive procedures	294 (69.02)	71 (16.67)	/	61 (14.32)	99 (23.24)	272 (63.85)	/	55 (12.91)	66 (15.49)	306 (71.83)	/	54 (12.68)	33 (7.75)	354 (83.10)	/	39 (9.15)
Blood transfusion or blood products	292 (68.54)	45 (10.56)	40 (9.39)	49 (11.50)	141 (33.10)	202 (47.42)	35 (8.22)	48 (11.26)	102 (23.94)	262 (61.50)	25 (5.87)	27 (8.69)	51 (11.97)	336 (78.87)	13 (3.05)	26 (6.11)
Antibiotics	329 (77.23)	25 (5.87)	34 (7.98)	38 (8.92)	200 (46.95)	171 (40.14)	34 (7.98)	21 (4.93)	211 (49.53)	162 (38.03)	32 (7.51)	21 (4.93)	68 (15.96)	321 (75.35)	6 (1.41)	31 (7.28)
Routine examinations	369 (86.62)	21 (4.93)	/	36 (8.45)	191 (44.83)	200 (46.95)	/	35 (8.22)	254 (59.62)	141 (33.10)	/	31 (7.28)	63 (14.79)	342 (80.28)	/	21 (4.93)
Analgesics (even if it causes confusion or shortens life)	/	/	/	/	/	/	/	/	298 (69.95)	38 (8.92)	46 (10.80)	44 (10.33)	286 (67.14)	86 (20.19)	13 (3.05)	41 (9.62)

### CHWs’ attitudes toward ADs

355 (83.33%) CHWs supported ADs, 356 (83.57%) participants agreed that AD will be increasingly applied in future applications and 355 (83.33%) thought that AD minimize patients’ suffering and dignity loss at the EOL stage. However, in terms of the application and training related issues, 322 (75.59%) CHWs felt that they lacked training on ADs (Table 5).

**Table 5:** CHWs’ attitudes toward ADs (*n* = 426).

Variables	*n* (%)
I support ADs	
Yes	355 (83.33)
No	71 (16.67)
ADs enhance patient autonomy	
Yes	352 (82.63)
No	74 (17.37)
Difficulty in implementing ADs without legal support	
Yes	374 (87.79)
No	52 (12.21)
ADs prompt doctors to consider withholding or withdrawing treatment	
Yes	349 (81.92)
No	77 (18.08)
ADs promote public acceptance of euthanasia	
Yes	307 (72.07)
No	119 (27.93)
ADs will be increasingly applied in future applications	
Yes	356 (83.57)
No	70 (16.43)
Everyone should have ADs for unexpected events	
Yes	356 (83.57)
No	70 (16.43)
ADs reduce emotional burden on family members	
Yes	363 (85.21)
No	63 (14.79)
Patients often change their choices for life-sustaining treatment in terminal illness	
Yes	361 (84.74)
No	65 (15.26)
Some people cannot make decisions for themselves even when conscious	
Yes	340 (79.81)
No	86 (20.19)
I will discuss ADs with patients in the future	
Yes	306 (71.83)
No	120 (28.17)
ADs avoid legal disputes over terminal patient medical decisions	
Yes	352 (82.63)
No	74 (17.37)
Prolonging life is more important than respecting patient’s wishes	
Yes	212 (49.77)
No	214 (50.23)
ADs reduce family disputes over treatment measures	
Yes	357 (83.80)
No	69 (16.20)
ADs minimize patient suffering and dignity loss	
Yes	355 (83.33)
No	71 (16.67)
Patients may feel they will not receive proper care if they sign ADs	
Yes	221 (51.88)
No	205 (48.12)
Nurses have a duty to advocate for patients when their wishes are not respected	
Yes	284 (66.67)
No	142 (33.33)
Patients should be informed of their condition to help make decisions	
Yes	341 (80.05)
No	85 (19.95)
Patients may sign ADs under pressure	
Yes	265 (62.21)
No	161 (37.79)
Even if analgesics shorten life, patients should receive aggressive pain relief	
Yes	325 (76.29)
No	101 (23.71)
Discussing ADs increases patient anxiety and depression	
Yes	259 (60.80)
No	167 (39.20)
My duty is to save lives, not to let them die	
Yes	321 (75.35)
No	105 (24.65)
Allowing patients to die is a crime	
Yes	148 (34.74)
No	278 (65.26)
After the patient loses consciousness, medical staff can make the best decisions without considering patient opinions	
Yes	158 (37.09)
No	268 (62.91)
Discussing ADs makes patients think I support giving up treatment	
Yes	201 (47.18)
No	225 (52.82)
Discussing ADs seems cold and heartless	
Yes	154 (36.15)
No	272 (63.85)
I think withdrawing treatment is immoral	
Yes	141 (33.10)
No	285 (66.90)
Patients lack sufficient knowledge to make AD decisions	
Yes	319 (74.88)
No	107 (25.12)
There are too many patients who need ADs	
Yes	258 (60.56)
No	168 (39.44)
If discussing ADs leads to patient giving up treatment, family will blame me	
Yes	260 (61.03)
No	166 (38.97)
Discussing ADs promotes suicide	
Yes	177 (41.55)
No	249 (58.45)
I’m afraid I might unintentionally suggest patients give up treatment, thus objectively reducing my workload	
Yes	201 (47.18)
No	225 (52.82)
I do not understand how ADs work	
Yes	264 (61.97)
No	162 (38.03)
Patients will think this is a hint that they are dying soon	
Yes	264 (61.97)
No	162 (38.03)
Discussing death might induce patients to choose death	
Yes	241 (56.57)
No	185 (43.43)
Life is extremely precious and should never be given up	
Yes	294 (69.01)
No	132 (30.99)
Patients make decisions more based on emotions than medical knowledge	
Yes	320 (75.12)
No	106 (24.88)
I might offend patients and their families	
Yes	168 (39.44)
No	258 (60.56)
I do not think the ADs training I’ve received is enough to answer patients’ questions	
Yes	322 (75.59)
No	104 (24.41)
Discussing ADs is not my duty	
Yes	218 (51.17)
No	208 (48.83)
Discussing ADs requires a lot of communication with patients, and I do not have enough time	
Yes	239 (56.10)
No	187 (43.90)
This might make patients feel like a burden	
Yes	230 (53.99)
No	196 (46.01)
I do not know where to get information on ADs	
Yes	312 (73.24)
No	114 (26.76)
ADs cross my psychological boundaries	
Yes	232 (54.46)
No	194 (45.54)
Giving up treatment is giving up hope	
Yes	220 (51.64)
No	206 (48.36)
I’ve received insufficient training on discussing death	
Yes	329 (77.23)
No	97 (22.77)
The emotional burden at work is too heavy for me to bear	
Yes	267 (62.68)
No	159 (37.32)
We should try to prolong terminal patients’ lives as much as possible, waiting for new therapies	
Yes	261 (61.27)
No	165 (38.73)
Doctors might no longer consider patients’ conditions thoroughly and rely solely on ADs	
Yes	191 (44.84)
No	235 (55.16)
Even if doctors think there’s hope, family members might still demand to terminate treatment according to ADs	
Yes	309 (72.54)
No	117 (27.46)

### Comparison of attitudes among CHWs with different characteristics

Utilizing “I Support for ADs” as the dependent variable, demographic data and values, the willingness to sign ADs and appoint medical proxy, organ donation intention as independent variables for conducting chi-square analysis. There were significant differences among 10 factors: education level, monthly income, belief in participating in medical decision-making, experience with prolonged treatment of unconscious terminal patients due to family’s reluctance to give up, considered own treatment choices in terminal illness, heard of ADs before, treatment measures, the willingness to sign ADs/appoint medical proxy/organ donation (*p* < 0.05). The results are presented in Table 6.

**Table 6:** Comparison of attitudes among CHWs with different characteristics (*n* = 426).

Variables	I support for ADs (*n*, %)		*χ* ^2^	*p*
	Yes	No		
Gender			0.558	0.455
Male	39 (10.99)	10 (14.08)		
Female	316 (89.01)	61 (85.92)		
Age, y			1.368	0.713
≤30	108 (30.42)	26 (36.62)		
31–40	156 (43.94)	28 (39.44)		
41–50	70 (19.72)	12 (16.90)		
≥51	21 (5.92)	5 (7.04)		
Ethnicity			0.000	1.000
Han	350 (98.59)	70 (98.59)		
Minority	5 (1.41)	1 (1.41)		
Occupation			0.776	0.378
Nurse	257 (72.39)	55 (77.46)		
Doctor	98 (27.61)	16 (22.54)		
Education level			6.735	0.041
Secondary technical school	14 (3.94)	5 (7.04)		
Junior college	102 (28.73)	29 (40.85)		
Bachelor’s degree	235 (66.20)	37 (52.11)		
Master’s degree or above	4 (1.13)	0 (0.00)		
Professional title			2.799	0.592
None	63 (17.75)	16 (22.54)		
Junior	116 (32.68)	27 (38.03)		
Intermediate	143 (40.28)	23 (32.39)		
Associate senior	24 (6.76)	3 (4.23)		
Senior	9 (2.54)	2 (2.82)		
Manager position			2.953	0.086
None	303 (85.35)	66 (92.96)		
Yes	52 (14.65)	5 (7.04)		
Work experience, y			3.544	0.617
<1	6 (1.69)	1 (1.41)		
1–5	52 (14.65)	14 (19.72)		
6–10	85 (23.94)	20 (28.17)		
11–15	95 (26.76)	19 (26.76)		
16–20	47 (13.24)	5 (7.04)		
>20	70 (19.72)	12 (16.90)		
Monthly income (Yuan)			8.152	0.043
<3,000	28 (7.89)	7 (9.86)		
3,000–5,000	180 (50.70)	47 (66.20)		
5,001–10,000	138 (38.87)	15 (21.13)		
>10,000	9 (2.54)	2 (2.82)		
Marital status			1.668	0.434
Unmarried	62 (17.46)	15 (21.13)		
Married	287 (80.85)	56 (78.87)		
Divorced	6 (1.69)	0 (0.00)		
Living status			1.522	0.467
Living alone	27 (7.61)	8 (11.27)		
Living with family	318 (89.58)	60 (84.51)		
Living with non-family	10 (2.82)	3 (4.23)		
Number of children				
None	84 (23.66)	21 (29.58)	1.115	0.573
One	195 (54.93)	36 (50.70)		
Two or more	76 (21.41)	14 (19.72)		
Religious belief			0.040	0.841
Yes	4 (1.13)	1 (1.41)		
No	351 (98.87)	70 (98.59)		
Self-rated health			1.442	0.837
Very good	88 (24.79)	15 (21.13)		
Good	117 (32.96)	24 (33.80)		
Average	139 (39.15)	31 (43.66)		
Poor	8 (2.25)	1 (1.41)		
Very poor	3 (0.85)	0 (0.00)		
History of serious illness affecting work/life			0.578	0.447
Yes	30 (8.45)	8 (11.27)		
No	325 (91.55)	63 (88.73)		
Experience of relatives’ painful death			0.008	0.931
Yes	192 (54.08)	38 (53.52)		
No	163 (45.92)	33 (46.48)		
Discussing life and death discomfort you			0.674	0.412
Yes	102 (28.73)	17 (23.94)		
No	253 (71.27)	54 (76.06)		
Belief in participating in medical decision-making			19.948	0.000
Yes	276 (77.75)	37 (52.11)		
No	79 (22.25)	34 (47.89)		
Views on destiny			2.925	0.232
Humans can control their destiny	164 (46.20)	25 (35.21)		
Do not want to plan ahead, wait and see	94 (26.48)	22 (30.99)		
Humans cannot change what will happen	97 (27.32)	24 (33.80)		
Difficulty in making medical decisions for self vs. seriously ill relatives			0.711	0.399
Self	107 (30.14)	25 (35.21)		
Relatives	248 (69.86)	46 (64.79)		
Importance of quality of life vs. length of life			2.669	0.102
Quality of life is more important	290 (81.69)	52 (73.24)		
Length of life is more important	65 (18.31)	19 (26.76)		
Presence of ethics committee in hospital			3.058	0.080
Yes	95 (26.76)	12 (16.90)		
No	260 (73.24)	59 (83.10)		
Experience with terminal patients			3.299	0.069
Yes	249 (70.14)	42 (59.15)		
No	106 (29.86)	29 (40.85)		
Discussed terminal medical choices with patients			3.341	0.068
Yes	91 (25.63)	11 (15.49)		
No	264 (74.37)	60 (84.51)		
Experience with prolonged treatment of unconscious terminal patients due to family’s reluctance to give up			4.795	0.029
Yes	229 (64.51)	36 (50.70)		
No	126 (35.49)	35 (49.30)		
Experience with family disputes over patient treatment choices			3.515	0.061
Yes	241 (67.89)	40 (56.34)		
No	114 (32.11)	31 (43.66)		
Considered own treatment choices in terminal illness			8.969	0.003
Yes	179 (50.42)	22 (30.99)		
No	176 (49.58)	49 (69.01)		
Heard of advance directives before			4.744	0.029
Yes	87 (24.51)	9 (12.68)		
No	268 (75.49)	62 (87.32)		
Impact of advance directives on terminal treatment aspects				
(1) Treatment measures			7.304	0.007
No impact	53 (14.93)	20 (28.17)		
Impact	302 (85.07)	51 (71.83)		
(2) Pain			0.157	0.692
No impact	93 (26.20)	17 (23.94)		
Impact	262 (73.80)	54 (76.06)		
(3) Terminal illness duration			0.407	0.523
No impact	92 (25.92)	21 (29.58)		
Impact	263 (74.08)	50 (70.42)		
Has your hospital provided any training on advance directives?			0.004	0.952
Yes	54 (15.21)	11 (15.49)		
No	301 (84.79)	60 (84.51)		
What kind of institution do you think should undertake the training for ADs?			0.372	0.83
University	58 (16.34)	11 (15.49)		
Hospital	214 (60.28)	41 (57.75)		
Academic organization	83 (23.38)	19 (26.76)		
Do you think you should sign an AD?			40.18	0.000
Yes	191 (53.80)	9 (12.68)		
No	66 (18.59)	25 (35.21)		
Do not know	98 (27.61)	37 (52.11)		
Will you appoint a medical proxy?			17.783	0.000
Yes	260 (73.24)	34 (47.89)		
No	95 (26.76)	37 (52.11)		
Will you donate organs?			8.442	0.004
Yes	182 (51.27)	23 (32.39)		
No	173 (48.73)	48 (67.61)		

### Binary logistic regression analysis of multiple factors related to the CHWs’ attitudes on ADs

Binary logistic regression was used to analyze the influencing factors of CHWs’ attitudes on ADs. The 10 factors that had a significant impact in the above analysis and knowledge scores on ADs were included in the logistic model. The results revealed that knowledge scores on ADs, education level, belief in participating in medical decision-making were independent risk factors for attitudes on ADs among CHWs (*p* < 0.05) (Table 7).

**Table 7:** Logistic regression analysis of the risk factors related to the CHWs’ attitudes.

Variables	*B*	Std. error	Wald *χ*^2^	*p*	OR (95% CI)
Constant	1.068	0.711	2.256	0.133	2.909 (0.722–11.721)
Knowledge scores on ADs	0.223	0.053	17.955	0	1.250 (1.128–1.386)
Education level	0.436	0.222	3.875	0.049	1.547 (1.002–2.389)
Belief in participating in medical decision-making	−0.952	0.282	11.357	0.001	0.386 (0.222–0.671)

## Discussion

This is the first study in China to evaluate the knowledge, intentions, and attitudes of CHWs in China toward ADs. Our research showed the mean knowledge score of CHWs regarding ADs (3.82 ± 2.85) was significantly lower than the findings (5.36 ± 2.23) by Cheng et al. (24), despite used the same knowledge scale. Nevertheless, a remarkable 83.33% of CHWs held a supportive attitude toward ADs, a proportion that aligns with the results of comparable studies (25–27). However, regarding the issue of “whether to sign an AD” in this study, the result (46.95%) was lower than that of other studies (28–30). Additionally, 31.69% adopted a wait-and-see stance, while 21.36% firmly decided not to sign. This contradiction can be attributed to several underlying concerns. A substantial 72.07% of CHWs believed that “ADs promote public acceptance of euthanasia,” and 60.8% agreed that “Discussing ADs increases patient anxiety and depression” (Table 5). Similar to the findings among Japanese community nurses (7), Chinese CHWs face a significant tension between respecting patients’ autonomy and maintaining family harmony (31). In this survey, 72.54% of CHWs expressed concerns that even if doctors believed there was hope for a patient’s improvement, families might request the termination of treatment based on ADs. This concern is well-founded, as China’s criminal law explicitly prohibits euthanasia, and many perceive the withdrawal of life-sustaining treatments through ADs as a form of euthanasia. In mainland China, there is currently a lack of strict differentiation among “ADs,” “LWs,” and “advance care planning” in practice (32). Moreover, the absence of specific legislation for ADs has led 87.79% of CHWs to identify legal obstacles as a major barrier to AD implementation. Compared with Japan, which promotes ADs indirectly through the “Medical Decision Support Law” (7), and South Korea, which relies on the draft of the “Dignity Death Law” (33), Chinese CHWs have a stronger perception of “difficulties in implementation without legal support” (13). This institutional gap has led many CHWs to default to the traditional “paternalistic” medical model when dealing with terminal patients, rather than actively promoting patients’ autonomous decision-making (31). To address this, it is essential to learn from international best practices, formulate a specialized law on EOL instructions, clarify the procedures for signing, modifying, and revoking such instructions, and ensure their effective implementation in actual medical decision-making processes. Additionally, in-depth research on AD-related legal issues should be strengthened to provide clear legal guidance for healthcare workers and reduce the legal risks associated with ADs in clinical practice.

This study also revealed that for every one-point increase in the ADs knowledge score, the likelihood of CHWs supporting ADs increased by 25% (OR: 1.250, 95% CI: 1.128–1.386). This finding strongly supports the KAP theory, which posits that knowledge serves as the foundation for attitude formation. In other words, the more CHWs understand ADs, the more they can recognize the importance of ADs in safeguarding patients’ autonomy and dignity at the EOL. Previous studies demonstrated that ADs knowledge scores among healthcare professionals, including CHWs, were significantly associated with more positive attitudes toward promoting and implementing advance care planning (34, 35). However, our study also highlighted a concerningly low rate of knowledge dissemination, with only 22.54% of CHWs having prior knowledge of ADs. Educational attainment emerged as another significant factor influencing CHWs’ attitudes toward ADs. CHWs with higher education levels were more likely to support ADs (OR: 1.547, 95% CI: 1.002–2.389), a result consistent with previous research (7, 36–38). Individuals with lower educational attainment often have limited familiarity with legal frameworks and encounter practical difficulties in understanding ADs, which hinders their ability to effectively engage with patients (35). Conversely, CHWs with higher levels of education typically exhibit stronger critical thinking skills and a deeper understanding of patients’ autonomy (35). However, considering that the overall educational background of CHWs in China is relatively low (13), promoting the concept of ADs remains a significant challenge. Due to the lack of legal clarity, ADs have not been incorporated into medical ethics education in China, resulting in limited understanding among CHWs. This situation is similar to that in countries such as Nigeria and Malaysia, which also lack a well-defined legal framework for ADs (39, 40). Moreover, the current focus of CHWs on providing chronic disease management and mental health support has led to a neglect of ADs training, despite the critical ethical and legal implications of ADs. Previous studies have suggested that ADs training and academic exchanges can effectively enhance the educational level and professional quality of CHWs (41, 42). Therefore, a two-step approach is necessary: first, legislative efforts should be prioritized to raise public awareness of ADs, and then ADs should be integrated into the medical education system.

Interestingly, this study found that the support level of CHWs for ADs was negatively correlated with the belief in participating in medical decision-making (OR: 0.386, 95% CI: 0.222–0.671), which contradicts the results of other studies (43, 44). In China, the paternalistic medical model, deeply rooted in Confucian culture, emphasizes the authority of physicians and family-based decision-making, thus limiting patient autonomy. Even within the current hierarchical healthcare policy framework, CHWs tend to maintain a pronounced paternalistic approach (45, 46). To overcome this cultural barrier, a combination of strategies is needed. On one hand, role-playing exercises and case studies can help CHWs mitigate cultural conflicts and enhance their respect for patient autonomy (34). On the other hand, policy incentives and comprehensive health education initiatives can play a crucial role in promoting patient participation and autonomy (45, 46).

## Limitations

This study acknowledges several limitations. Firstly, the use of convenience sampling may limit the generalizability of our findings to wider populations. Secondly, the cross-sectional design of the study prevents definitive inferences about causality. Thirdly, compared to face-to-face surveys, online surveys are more prone to result in information bias. Additionally, potential confounding factors, such as religious beliefs and other unmeasured variables, were not fully considered in the analysis, which could have impacted the results. Despite these limitations, our study has its strengths. It utilized a large sample size, and it is the first study of its kind conducted on knowledge about ADs among CHWs in China.

## Conclusion

In conclusion, this study demonstrated that although the knowldedge level of CHWs regarding ADs is relatively low, their attitude toward ADs is predominantly positive. The main factors influencing their attitudes include knowledge level, education level, and belief in participating in medical decision-making. To promote the implementation of ADs in China, the following recommendations are put forward: (1) Accelerate the legislative process for ADs and clearly define the roles and responsibilities of CHWs in EOL care; (2) Develop a stratified training system based on the KAP theory and incorporate ADs knowledge into the compulsory modules of continuing medical education; (3) Use role-playing and case analysis to help CHWs overcome “paternalistic” thinking and strengthen their respect for patients’ autonomy.

## Data Availability

The original contributions presented in the study are included in the article/supplementary material, further inquiries can be directed to the corresponding author.
